# Anion order in perovskite oxynitrides AMO_2_N (A = Ba, Sr, Ca; M = Ta, Nb): a first-principles based investigation†

**DOI:** 10.1039/d0ra03681a

**Published:** 2020-06-25

**Authors:** Xi Xu, Hong Jiang

**Affiliations:** Beijing National Laboratory for Molecular Sciences, College of Chemistry and Molecular Engineering, Peking University 100871 Beijing China jianghchem@pku.edu.cn

## Abstract

Perovskite-type oxynitrides have attracted a lot of research interest as emerging functional materials with promising wide applications. The ordering of O/N anions in perovskite oxynitrides plays an important role in determining their physical properties, while it is still challenging to characterize the actual anion order in a particular material and understand the underlying physics. In this work, we have investigated anion order in a series of perovskite oxynitrides AMO_2_N (A = Ba, Sr, Ca; M = Ta, Nb) through first-principles calculations and the cluster-expansion-model-based Monte Carlo simulations. In terms of cluster correlation functions, it can be explicitly demonstrated that short-range anion order is present in all these perovskite oxynitrides. In addition, the anion order varies with the temperature of thermal equilibrium and depends on the cation type. Special quasi-ordered structures are then constructed as representative structures by taking the calculated anion order at finite temperature into consideration and their band gaps and dielectric tensors are predicted by first-principles calculations and compared to experimental values.

## Abstract 2

Anion order in perovskite oxynitrides is investigated by a combination of first-principles calculations, cluster expansion method and Monte Carlo simulations.

## Graphical abstract

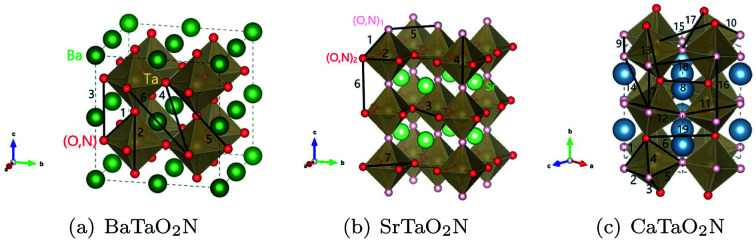

## 1 Introduction

Transition metal oxynitrides combine the advantages of the stability of oxides and the suitable band gaps of nitrides and therefore show great potential in photocatalytic and photoelectric applications.^1–4^ Typical examples are N-doped TiO_2_,^5^ (GaN)_1−*x*_(ZnO)_*x*_ solid solutions^6^ and LaTiO_2_N.^7^ In particular, because of the flexibility of the perovskite structure, perovskite-type oxynitrides have been productively explored and have evolved into a large family of materials^4,8^ with important potential applications.^1,4,9–11^ Bulk BaTaO_2_N and SrTaO_2_N are reported to have large dielectric constants and are promising to be used as working materials for capacitors.^12^ MSi_2_O_2−*δ*_N_2+2/3*δ*_:Eu^2+^ (M = Ba, Sr, Ca) oxynitrides are promising conversion phosphors for light-emitting diodes.^13^ Large coupled magneto-responses are found in EuNbO_2_N.^14^ In addition, many perovskite oxynitrides have been found to be active photo-catalysts for visible-light-driven water splitting because of their suitable band gaps and absolute band positions.^2,11,15,15–18^

Except for special cases such as β-TaON, in which O and N atoms are orderly arranged,^19^ most oxynitrides exhibit a disordered distribution of O and N atoms according to neutron diffraction experiments (for example, see ref. 20). From the perspective of solid state chemistry, the disordering tendency in oxynitrides can be attributed to two facts: (1) the ionic radii of O^2−^ and N^3−^, which are 1.38 and 1.46 Å, respectively, are quite close, and (2) the chemical bonding between O/N and neighboring high-valent metals has substantial covalent characters, which can lead to similar M–O and M–N bond lengths and also similar effective charges of O and N.^21^ Nevertheless, short-range order or partial order can still exist in consistency with a long-range disordered model fitted from diffraction data, as suggested by ref. 22 and reviewed by ref. 21. The O/N anion order is predicted to have a significant influence on properties such as the band gap based on first-principles calculations on supercells of oxynitrides with specific symmetries.^23–28^ However, it is still quite challenging to determine the actual anion order in a particular material. In our previous work of BaTaO_2_N,^29^ we showcased a general scheme to investigate anion order in oxynitrides by combining first-principles calculations and cluster-expansion-based Monte Carlo (MC) simulations. In this work, we apply the same approach to a series of perovskite oxynitrides AMO_2_N (A = Ba, Sr, Ca; M = Ta, Nb). We demonstrate how anion order can be influenced to some extent by the type of cations (A or M) or the temperature of thermal equilibrium. Then anion order is taken into consideration for building representative structures to calculate the electronic and dielectric properties of these materials.

The rest of this paper is organized as follows. In Section 2 the computational details of this work are presented. In Section 3 the cluster expansion models obtained for these perovskite oxynitrides are first discussed, and then the anion order in different oxynitrides is addressed in terms of cluster correlation functions; electronic and dielectric properties of these perovskite oxynitrides are then investigated by using representative structures that have taken short-range anion order into account. In Section 4, we close the paper by summarizing our main findings.

## 2 Theoretical method and computational details

In this section we give a brief introduction of the theoretical approach used in this work.^29^ We adopt essentially the same procedures for each material considered in this work. Firstly, a cluster expansion model is built by fitting the energies of about one hundred different configurations calculated by density-functional theory (DFT). Secondly, Monte Carlo simulations based on the cluster expansion model are performed to reveal possible phase transitions and obtain a quantitative characterization of anion order at different temperatures. Finally, special quasi-ordered structures (SQoSs),^29–31^ which take the short-range order at finite temperature into consideration, are built as representative structures to obtain electronic and dielectric properties.

We consider six typical perovskite oxynitrides AMO_2_N (A = Ba, Sr, Ca; M = Ta, Nb) in this work that have been intensively investigated experimentally.^2,8,12,22,32–43^ The experimental crystal structures from ref. 12, as collected in Table 1, are used as the underlying lattice to construct cluster expansion models. The lowered symmetry of tetragonal/orthorhombic phases is mainly caused by the tilting of M(O,N)_6_ octahedra and leads to a significant increase of the number of symmetrically distinct clusters for a given cutoff diameter.

**Table 1:** The space group (SG), experimental (from ref. 12) and calculated (averaged over randomly generated configurations) lattice parameters (in unit of Angstrom) of AMO_2_N (A = Ba, Sr, Ca; M = Ta, Nb). The right columns collect the size of training set *N*_s_, the number of clusters considered, the root-mean-squared derivation (RMSD) and the leave-one-out cross-validation (LOOCV) scores (in unit of meV per atom) of optimal cluster expansion models for DFT-calculated ground energies

System	SG	Expt.			Calc.			*N*_s_	*N*_c_	RMSD	LOOCV
		*a*	*b*	*c*	*a*	*b*	*c*				
BaTaO_2_N	*Pm*3*m*	4.113	4.113	4.113	4.106	4.108	4.107	125	8	1.2	1.3
SrTaO_2_N	*I*4/*mcm*	5.703	5.703	8.054	5.699	5.670	8.022	111	10	1.9	2.1
CaTaO_2_N	*Pnma*	5.619	7.893	5.549	5.622	7.860	5.522	111	37	1.0	1.2
BaNbO_2_N	*Pm*3*m*	4.128	4.128	4.128	4.118	4.121	4.123	109	8	3.2	3.5
SrNbO_2_N	*I*4/*mcm*	5.710	5.710	8.104	5.737	5.736	8.075	90	10	3.0	3.4
CaNbO_2_N	*Pnma*	5.641	7.907	5.555	5.634	7.863	5.517	97	37	1.4	1.9

For each system, we generate about 100 configurations with randomly distributed O/N atoms within a 40-atom supercell. All configurations are distinct in energy to rule out the possibility that two randomly generated configurations are symmetrically equivalent. Kohn–Sham DFT, as implemented in Vienna Ab initio Simulation Pack^44,45^ (VASP version 5.4.4), is then used to obtain the total energy of each configuration in their respectively fully relaxed geometry. Valence-core interactions are treated by the projector augmented wave (PAW) method,^46^ in which 3s and 3p states of Ca, 4s and 4p states of Sr, 5s and 5p states of Ba, 4p states of Nb and 5p states of Ta are treated as valence states. The energy cutoff of the plane wave basis is set to be 520 eV. DFT in the Perdew–Burke–Ernzerhof-for-solids (PBEsol)^47^ density-functional approximation is used to obtain a better description of equilibrium lattice properties.^48,49^ The Brillouin zone is sampled by the density of 1000 *k* points per reciprocal atom. The energy convergence criterion for self-consistent field iterations is set to be 10^−5^ eV. Both the unit cell parameters and internal atomic positions are simultaneously relaxed by the conjugate gradient algorithm until the energy difference between two ionic steps is less than 10^−4^ eV. In Table 1 we collected the calculated lattice constants (*a*, *b* and *c*) of AMO_2_N obtained by averaging over different O/N occupation configurations, which show a quite good agreement with experimental values.

The calculated DFT energies of randomly generated configurations are then collected and fitted linearly to cluster expansion models by the least-squares fitting algorithm with the help of Alloy Theoretic Automated Toolkit (ATAT).^50,51^ The essence of the cluster expansion approach is to express the locally relaxed energy of any specific configuration *σ* of a binary alloy A_1−*x*_B_*x*_ by a sum of interactions of clusters (see ref. 52–54 for a more systematic treatment of general multi-component and multi-sublattice cases)1
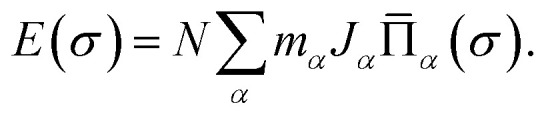
Here *α* denotes a cluster corresponding to a set of sites of the underlying lattice with a given topology, *N* is the number of mixed occupied lattice sites, *m*_*α*_ is the number of symmetry-related clusters per site, *J*_*α*_ is termed as effective cluster interaction (ECI), and 
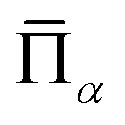
 is the lattice-averaged cluster function, which is the product of pseudo-spin variables (+1 for A atom and −1 for B atom) in corresponding lattice sites and averages over all symmetrically equivalent clusters2
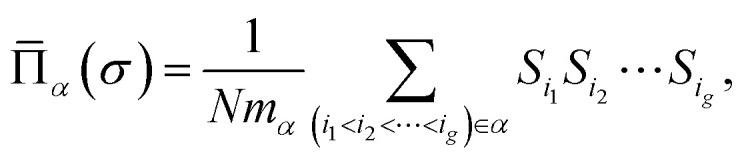
with *g* denoting the number of sites in the *α*-cluster. We have paid special attention to optimize the choice of clusters for the construction of models. The accuracy of a given cluster expansion model is characterized by the leave-one-out cross-validation (LOOCV) score,^50^3
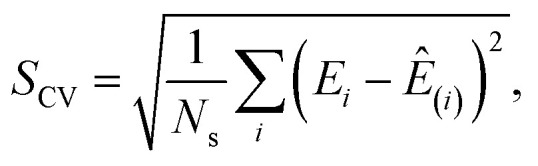
where *N*_s_ is the number of configurations in the training set, *E*_*i*_ is the directly calculated DFT energy of the *i*-th structure, and *Ê*_(*i*)_ is the fitted energy of *i*-th configuration using the cluster expansion model built with *E*_*i*_ excluded from the fitting set. We built the optimal CE model by taking a hierarchical approach that converges with respect to two-body, three-body and four-body clusters in turn as used in our previous work.^29^ Then the learning curves are used to check its convergence with respect to the size of training set.

The cluster expansion model constructed as above is then served as the model Hamiltonian for Monte Carlo (MC) simulations to sample the canonical ensemble of a large supercell of perovskite oxynitrides to evaluate cluster correlation functions (CCFs),4
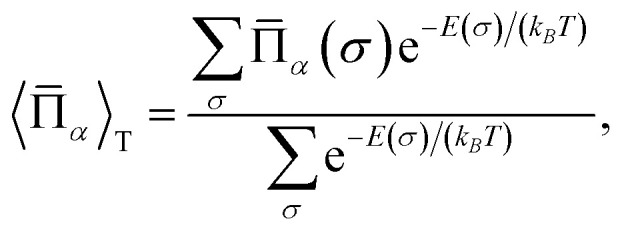
which provides a statistic description of the distribution and coupling of O and N occupation. The equilibration time and sampling time for the MC simulations are automatically determined by the algorithm proposed by ref. 55 and the target tolerance for the statistically averaged energy is set to be 1 × 10^−5^ eV, which has been tested to be adequate for the numerical convergence of CCFs.

Special quasi-ordered structures (SQoSs) are then generated by taking the above calculated cluster correlation functions into account. The cluster functions of SQoSs to be matched are in accordance with the optimal cluster expansion models. SQoSs are fully relaxed by the same parameters described above, while for the band structure properties we use the screened hybrid functional Heyd–Scuseria–Ernzerhof (HSE06)^56,57^ to overcome the band gap problem of local density approximation (LDA) or generalized gradient approximation (GGA).^58^ Using SQoSs as representative structures, we further investigate dielectric properties of oxynitrides by calculating Born effective charge tensors, high-frequency dielectric tensors, and dynamical matrices within the framework of density functional perturbation theory (DFPT).^59^

## 3 Results and discussion

### 3.1 Cluster expansion models

**Fig. 1:**
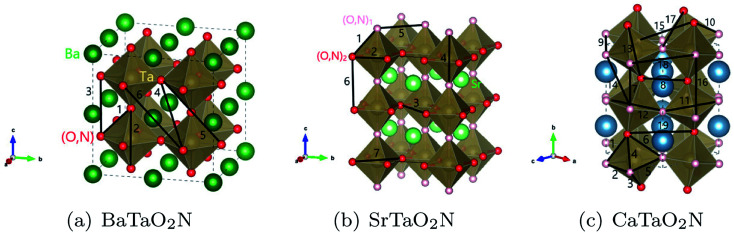
Two-body clusters for (a) BaTaO_2_N, (b) SrTaO_2_N and (c) CaTaO_2_N. The clusters are the same for tantalum oxynitrides and its niobium counterpart.

**Fig. 2:**
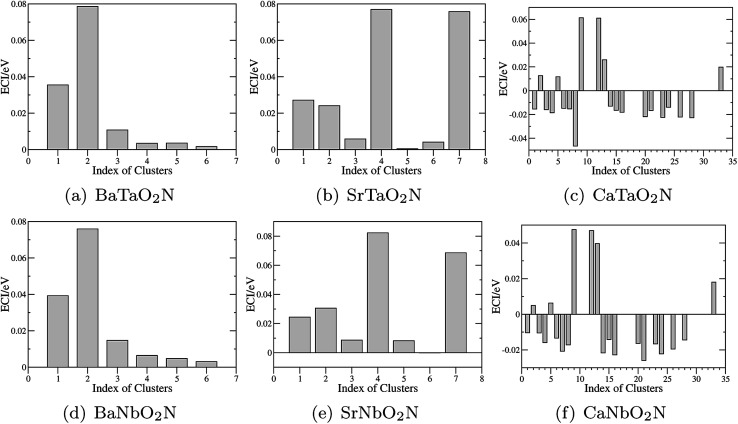
Effective cluster interactions (ECIs) of two-body clusters. The indices of clusters are the same as those in Fig. 1.

The optimization of cluster expansion models for the configuration dependence of the energy is based on the same procedure employed in our previous work on BaTaO_2_N.^29^ The results for the cluster optimization are presented in Fig. S1 in ESI.† It can be seen that short-range two-body clusters play dominant roles and the inclusion of higher-order clusters makes little difference. For each system, the training set is composed of about 100 configurations. As shown in Fig. S2 in ESI,† the training scores and test scores reach a plateau with the last two sizes of the training set, indicating the cluster expansion models could not benefit from the addition of more training configurations. Besides, the final gaps between the training scores and test scores are less than 0.5 meV, which means the predicting of new configurations is almost as accurate as fitting. Therefore, we conclude that the size of about 100 configurations is adequate for the construction of cluster expansion models for these perovskite oxynitrides. The root-mean-squared deviation (RMSD) and the LOOCV scores of optimized cluster expansion models are listed in Table 1, which fall into the range of 1 to 4 meV per atom and also indicate a good fitting and predictive power of corresponding cluster expansion models. Niobium and tantalum oxynitrides with the same alkaline earth element are isostructural,^12^ and the final cluster expansion models for them are composed of essentially the same set of clusters. Two-body clusters present in the CE models for the cubic (for BaTaO_2_N and BaNbO_2_N), tetragonal (for SrTaO_2_N and SrNbO_2_N), and orthorhombic (for CaTaO_2_N and CaNbO_2_N) structures are illustrated in Fig. 1. The corresponding ECIs in the CE models of AMO_2_N (A = Ba, Sr, Ca; M = Ta, Nb) are shown in Fig. 2. We note that the number of clusters in the CE model for CaTaO_2_N is significantly larger than that for BaTaO_2_N and SrTaO_2_N because of the significantly lowered symmetry compared to the ideal cubic perovskite structure.

Several features are noticeable from the ECIs of different perovskite oxynitrides. Firstly, niobium and tantalum oxynitrides with the same alkaline earth elements have similar ECIs, clearly indicating the close resemblance in the nature of chemical bonding in these two series of compounds. Secondly, for BaTaO_2_N, BaNbO_2_N, SrTaO_2_N and SrNbO_2_N, the two-body clusters corresponding to the edge and diagonal of Ta(O,N)_6_ octahedron (cluster 1 and 2 for BaTaO_2_N and BaNbO_2_N, 1, 2, 4, and 7 for SrTaO_2_N and SrNbO_2_N) are dominant and have positive ECIs, and the contributions of long-distance two-body clusters decay quickly as the cluster diameter increases, which implies that short-range order will mainly occur within the Ta(O,N)_6_ octahedron, as we have found for BaTaO_2_N previously.^60^ The positiveness of these two-body ECIs indicates O–N pairing in corresponding sites are energetically preferred than O–O or N–N pairing, which indicates that these oxynitrides would be stable against phase separation at low temperature. In contrast, the situations in CaTaO_2_N and CaNbO_2_N are much more complicated. There are a much larger number of two-body clusters with sizable ECIs and most of them are negative, which indicates that CaTaO_2_N and CaNbO_2_N could possibly undergo phase separation at low temperature, and one can expect more complicated short-range ordering behavior at finite temperature.

### 3.2 Anion ordering

The anion order in oxynitrides plays a crucial role in determining the physical and chemical properties of oxynitrides.^21^ To quantitatively characterize the anion order in the perovskite oxynitrides, we evaluate the cluster correlation functions by conducting the Monte Carlo simulation based on the cluster expansion model obtained above. The anion order in BaTaO_2_N was already presented in details in our previous work.^60^ As the anion order in BaNbO_2_N is very similar to that in BaTaO_2_N, as clearly seen by CCFs corresponding to major two-body clusters in BaTaO_2_N and BaNbO_2_N in Fig. S4 in ESI,† we will mainly discuss the anion order in SrTaO_2_N, SrNbO_2_N, CaTaO_2_N and CaNbO_2_N.

#### 3.2.1 SrTaO_2_N and SrNbO_2_N

**Fig. 3:**
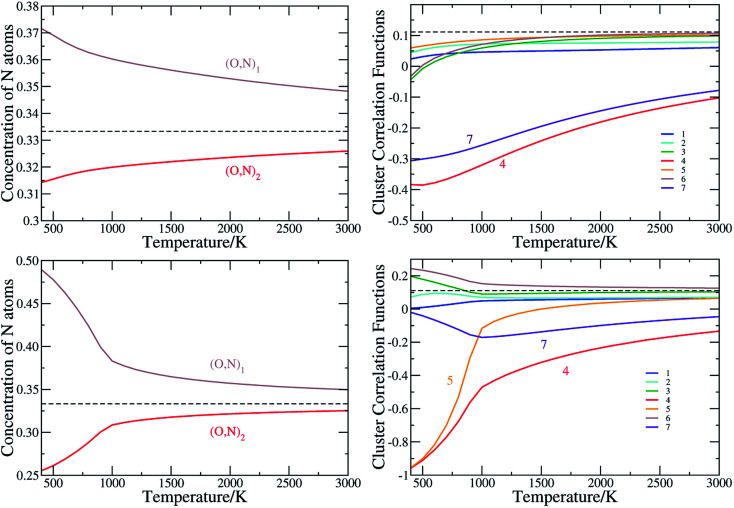
The N occupation concentration at two nonequivalent sites (left panels) and (right panels), and the cluster correlation functions for two-body clusters of SrTaO_2_N (upper panels) and SrNbO_2_N(lower panels). Clusters are illustrated in Fig. 1(b). The values for the concentration and cluster correlation functions of two-body clusters of the totally random state are shown as dashed lines.

For SrTaO_2_N, which takes a tetragonal crystal structure with the space group of *I*4*mcm* at room temperature,^12,61^ there are two symmetrically distinct sites for the mixed occupation of O and N atoms, labeled as (O,N)_1_ and (O,N)_2_ respectively in Fig. 1(b). The statistically averaged concentration of N atoms on these two sites as a function of the temperature is shown in Fig. 3(a). If O and N atoms are distributed fully randomly, the concentration of N atoms takes the value of 1/3. It can be seen that the (O,N)_1_ sites are always more likely to be occupied by N atoms than the (O,N)_2_ sites. Nevertheless, the difference is less than 0.07 and decreases monotonously as the temperature rises. This is consistent with the so-called pseudo-cubic structural model of SrTaO_2_N fitted in diffraction experiments.^12,61^ CCFs for two-body clusters of SrTaO_2_N are presented in Fig. 3(b). The CCFs of the 4th and 7th two-body clusters, corresponding to the diagonals of the Ta(O,N)_6_ octahedron, always deviate significantly from the totally random value 1/9 even at very high temperature and those of the 1st and 2nd two-body clusters, corresponding to the edge of the Ta(O,N)_6_ octahedron, also show significant deviation. In contrast, the CCFs corresponding to inter-octahedron two-body clusters are very close to the value of the fully random state. It indicates that the short-range anion order mainly exists within the Ta(O,N)_6_ octahedra, qualitatively similar to that in BaTaO_2_N. The feature that the anion ordering of SrTaO_2_N is robust at high temperatures is supported by the experimental results from variable-temperature powder diffraction.^62^

We have seen in Section 3.1 that ECIs for SrNbO_2_N are qualitatively similar to those of SrTaO_2_N, and therefore one would expect similar anion ordering features in the two systems. However, there are some significant differences in the CCFs of SrTaO_2_N and SrNbO_2_N, due to the subtle differences in the ECIs of two systems. The difference in the occupation of two nonequivalent sites is significantly larger in SrNbO_2_N than that in SrTaO_2_N, as clearly shown in Fig. 3(c). At *T* = 300 K, the concentrations of N atoms at the (O,N)_1_ and (O,N)_2_ sites are close to 0.5 and 0.25, respectively. Above the temperature of about 900 K, the CCFs of SrNbO_2_N are very similar to those of SrTaO_2_N, while below 900 K they differ significantly. In particular, the CCFs of the 4th and 5th two-body clusters are close to −1.0 at 300 K, which indicates that if one site is occupied by N then the other site in the cluster is almost definitely occupied by O, and *vice versa*. Therefore there is a strong correlation in the occupation of the two nearest inter-Ta(O,N)_6_ octahedra sites, which can introduce further long-range correlations. As shown in Fig. S3(c) in ESI,† the variance of the energy as a function of temperature exhibits a peak at around 900 K, indicating a second-order phase transition, which is not observed in SrTaO_2_N.

We note that the anion order observed here in SrTaO_2_N and SrNbO_2_N does not fully agree with the partial anion order model proposed in ref. 22, in which it is suggested, based on neutron diffraction experiments, that at low temperatures M–N chains are disorderly distributed in two-dimensional layers rather than the whole three-dimensional space. Further experiments confirm that such two-dimensional anion ordering is robust with respect to the temperature.^62^ The local *cis* configuration is supposed to maximize the covalency of M–O/N bonds,^63^ and two-dimensional –N–M–N– chains could therefore gain great energetic stability from conjugate effect, just as that in large π bonds in plane molecules. Such a conjugate effect, however, is a many-body effect and could hardly be captured by truncated cluster expansion models with relatively small super-cells as the training set.^64^ This is a possible reason for why our calculated anion order does not agree with that determined experimentally.

#### 3.2.2 CaTaO_2_N and CaNbO_2_N

**Fig. 4:**
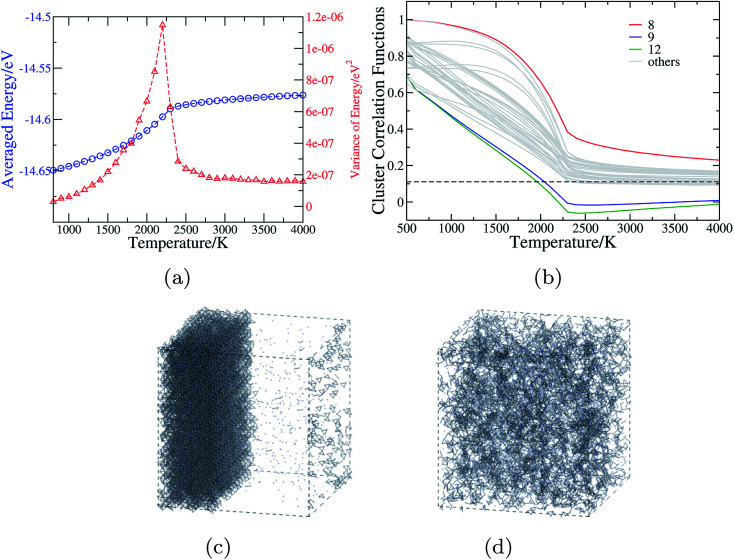
The anion order in CaTaO_2_N revealed by Monte Carlo simulation. (a) The mean and variance of the energy in the MC simulations as a function of temperature. (b) Cluster correlation functions corresponding to two-body clusters as illustrated in Fig. 1(c), with those of 8th, 9th and 12th clusters explicitly denoted and the dashed line indicating the value corresponding to the totally random state. (c) and (d) The snapshots from MC simulations of 15 × 15 × 11 supercell of CaTaO_2_N at *T* = 1000 K and *T* = 3000 K, respectively (the small blue balls stand for N atoms, and Ca, Ta and O are omitted for sake of clarity; when two N atoms are in the adjacent positions, they are connected by a gray bond).

As discussed in Section 3.1, ECIs of the cluster expansion models for CaTaO_2_N and CaNbO_2_N are significantly different from those for other perovskite oxynitrides in the sense that the CE models for these two systems contain a much larger number of clusters, and that many of ECIs are negative. Consequently, the anion order in CaTaO_2_N and CaNbO_2_N can be expected to be much more complicated. The statistically averaged energy and its variance as a function of temperature obtained from the MC simulations of a large supercell of CaTaO_2_N are presented in Fig. 4(a). The variance remarkably shows a distinct “discontinuity” at around 2300 K, which indicates a phase transition at that temperature. The MC snapshots taken below and above the transition temperature (Fig. 4(c) and (d)) indicates that CaTaO_2_N is stable only above the critical temperature and it experiences a phase separation below the critical temperature with one phase O-aggregated and the other one N-aggregated. The composition of the O-aggregated phase is close to Ca_2_Ta_2_O_7_, which turns up as a competing phase in the process of experimental preparation of CaTaO_2_N.^12^ The N-aggregated phase has the composition close to Ca_3_Ta_3_N_7_. In reality, this may not be a stable phase and further decomposition into Ca_3_N_2_ and Ta_3_N_5_ could take place, which, however, can not be captured due to the limitation of the lattice model used in our MC simulations. The critical temperature predicted by the MC simulation is as high as 2200 K, which is consistent with the experimental finding that Ca_2_Ta_2_O_7_ can not be nitridated into CaTaO_2_N even after 240 h at 1373 K.^12^ On the other hand, since our MC simulations are based on the lattice model that considers only the configuration degrees of freedom, and ignores all other contributions, including especially those of lattice vibrations, the critical temperature is likely to be overestimated.^65^

Fig. 4(b) shows the CCFs of two-body clusters in CaTaO_2_N. It can be observed that most two-body clusters tend to the random occupation of O/N above the critical temperature, except for the 8th, the 9th and the 12th ones. This is consistent with their large ECIs in Fig. 2(c) and indicates the existence of strong anion order of O/N occupation in corresponding sites. The values of their CCFs differ significantly from the value of the fully random state even at very high temperatures. As shown in Fig. 1(c), the 8th and the 9th clusters are two sites between two nearest neighboring Ta(O,N)_6_ octahedra, which indicates that the anion order in CaTaO_2_N exceeds octahedra and is much more complicated than that in BaTaO_2_N and SrTaO_2_N.

The CCFs of CaNbO_2_N are shown in Fig. S4(c) in ESI,† and the overall features are very similar to those of CaTaO_2_N, except that the predicted critical temperature is about 1500 K. Therefore one can expect that CaNbO_2_N is likely to decompose into a N-aggregated phase and Ca_2_Nb_2_O_7_ below the critical temperature. The lower critical temperature of CaNbO_2_N than that of CaTaO_2_N (2200 K) is consistent with the experimental finding that CaNbO_2_N can be directly synthesized by the ammonolysis of Ca_2_Nb_2_O_7_ at 1003 K for 80 h while CaTaO_2_N can not.^12^ We note again that the calculated critical temperature is probably over-estimated by about several hundreds Kelvin because of the neglect of other degrees of freedom in the MC simulations.^65^

### 3.3 Band gaps and dielectric properties

Since the perovskite oxynitrides considered in this work all exhibit significant short-range anion order, we adopt the SQoS method to build representative structures by taking the anion order into consideration.^29,31,67^ For CaTaO_2_N and CaNbO_2_N, oxynitrides can only come into formation above the critical temperatures according to preceding MC simulations, therefore we choose CCFs at *T* = 3000 K as the target for the construction of SQoS. For other oxynitrides, we choose CCFs at *T* = 1300 K to be matched because it is close to their synthesis temperature.^12^ First-principles calculations are performed with these representative structures. The calculated band gaps and dielectric properties are collected in Table 2.

**Table 2:** Band gaps (by PBEsol and HSE06), reduced Born effective charges 
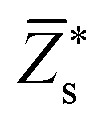
 and dielectric constants calculated by the PBEsol-based DFPT, using the 40-atoms SQoS for AMO_2_N (A = Ba, Sr, Ca, M = Ta, Nb). For the band gaps, we also show the HSE06 results calculated by using the most stable configuration (MSC). Experimental results for band gaps and dielectric constants are also collected for comparison. It should be noted that experimental dielectric constants are measured by using thin film samples, and therefore can be affected by epitaxial stress of substrates or interfacial effects

		BaTaO_2_N	SrTaO_2_N	CaTaO_2_N	BaNbO_2_N	SrNbO_2_N	CaNbO_2_N
*E*_g_	PBEsol	1.23	1.44	1.39	0.89	1.27	1.43
	HSE06	2.19	2.53	2.46	1.82	2.33	2.47
	HSE06(MSC)	2.16	2.08	2.85	1.58	1.65	2.37
	Expt.	1.8^a^, 2.0^b^	2.1^a^, 2.2^b^	2.4^a^, 2.6^b^	1.7^c^, 1.8^a^	1.8^c^, 1.9^a^	2.0^c^, 2.1^a^
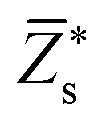	A	2.75	2.52	2.43	2.76	2.53	2.44
	M	8.19	8.15	7.70	7.91	7.92	7.57
	O	−3.37	−3.36	−3.09	−3.32	−3.34	−3.11
	N	−4.20	−3.94	−3.94	−4.03	−3.77	−3.79
*ε*	*ε* ^∞^	7.2	6.8	6.8	7.5	7.4	7.3
	*ε*_phonon_	120.9	93.5	54.3	92.3	103.7	53.6
	*ε*_total_	128.1	100.3	61.1	99.8	111.1	60.9
	*ε*_Expt._	220^d^	80^e^	30^f^	—	—	—

a From ref. 12.

b From ref. 34.

c From ref. 66.

d From ref. 37.

e From ref. 27.

f From ref. 12.

The PBEsol band gaps are about 1 eV smaller than the HSE06 ones for all these representative structures and the latter are in much better agreement with experiment. Therefore, in following we focus on the comparison of the HSE06 band gaps and experimental values. The calculated band gaps for SQoS are generally larger than corresponding experimental values. The averaged error is about 0.3 eV, which falls into the range of typical errors of HSE06 for the prediction of band gaps of semiconductors.^58^ One should also note that there is about 0.1–0.2 eV uncertainty in the experimentally measured values of the band gap.^12,34,66^ We also collect in Table 2 the HSE06 band gaps calculated with the structures of the most stable configuration (MSC). Overall the band gaps from SQoS and MSC structures are quite close, indicating that the configuration-dependence of the band gaps in these perovskite oxynitrides is not strong compared to that in alloyed oxynitride semiconductors like (ZnO)_1−*x*_(GaN)_*x*_.^60^

Dielectric properties of BaTaO_2_N have been discussed in our previous work^29^ and here we will focus on the comparison of dielectric properties between different perovskite oxynitrides. Huge dielectric constants over thousands were reported for ceramic samples of BaTaO_2_N and SrTaO_2_N,^12^ which, however, may be mainly extrinsic rather than intrinsic.^68,69^ Dielectric constants of thin films for BaTaO_2_N and SrTaO_2_N are about 220 (ref. 37) and 80 (ref. 27) respectively, which may be closer to their intrinsic values but still could be affected by epitaxial stress of substrates or interfacial effects. In contrast, the dielectric constant of CaTaO_2_N is only about 30 and no information about dielectric properties of all Nb analogue is available.^12^

The calculated dielectric properties of representative structures of AMO_2_N are collected in Table 2. The low symmetry of SQoS leads to an increase in the number of nonequivalent atoms in the supercell and anisotropy of Born effective charge tensors. In order to make a comparison between these oxynitrides, the averaged Born effective charge of the same type of atoms are presented.^29^ The nominal ionic charges for A (Ba, Sr, Ca), M (Ta, Nb), O and N atoms are +2, +5, −2 and −3 respectively, while their reduced Born effective charges are greater in these perovskite oxynitrides, particularly for BaTaO_2_N. The large Born effective charges indicate a mixed ionic-covalent nature of M–O/N bonds,^70^ which is consistent with the energetic preference of the *cis* configuration against the *trans*. There are no imaginary frequencies for the zone-center phonons of these representative structures, indicating that they are dynamically stable. Phonons in BaTaO_2_N, SrTaO_2_N, BaNbO_2_N and SrNbO_2_N are softer than those in CaTaO_2_N and CaNbO_2_N (see Fig. S5 in ESI†), which accounts for the difference in their calculated dielectric constants since soft modes make dominant contributions. The order of the values of the calculated dielectric constants of BaTaO_2_N, SrTaO_2_N and CaTaO_2_N is consistent with that of their experimental values. However, only the theoretical value for SrTaO_2_N is close to its experimental value 80. There is a severe underestimation for BaTaO_2_N while an overestimation for CaTaO_2_N. All Nb oxynitrides are predicted to have similar dielectric properties as corresponding Ta analogues. There are several factors that may underlie the discrepancies between calculated and experimental values of dielectric constants. The currently available experimental values of dielectric constants of these compounds may not be intrinsic ones due to the sample quality and other extrinsic factors that we have mentioned. On the other hand, there are also limitations in our theoretical treatments.^29^ First of all, we used the SQoS method to model O/N disordering, which may not be fully adequate to treat partial configuration disorder in these materials. Secondly, as discussed in ref. 71, DFPT calculation of dielectric constants exhibits a mean averaged derivation from experiment of about 16.2% for ordered structures. Perovskite oxynitrides are disordered systems and the situations can be more complicated. Thirdly, the contribution of anharmonicity to dielectric constants in these oxynitrides is not considered, and can be important since many perovskite materials exhibit ferro-electric behaviors.^70^ Obviously, more efforts are needed, both experimentally and theoretically, to obtain more thorough understanding of dielectric properties of perovskite oxynitrides.

## 4 Conclusions

To summarize, we have investigated anion order in perovskite-type oxynitrides AMO_2_N (A = Ba, Sr, Ca; M = Ta, Nb) through a combination of first-principles calculations, the cluster expansion method and Monte Carlo simulations. We built cluster expansion models for these perovskite oxynitrides respectively based on DFT calculations. We found that the effective cluster interactions of niobium oxynitrides are generally similar to those of their tantalum counterparts, but there are still noticeable differences that can result in significant differences in their anion order behaviors. BaTaO_2_N, SrTaO_2_N, BaNbO_2_N and SrNbO_2_N prefer *cis* configurations at low temperatures, while CaTaO_2_N and CaNbO_2_N exhibits the phase separation tendency at low temperature and become stoichiometrically stable only at high temperatures. Thus the anion order in perovskite oxynitrides can be significantly influenced by the composition and varies as a function of temperature. We build SQoS as representative structures for these perovskite oxynitrides with short-range order taken into account based on the calculated cluster correlation functions from Monte Carlo simulation. The calculated band gaps by the hybrid functional HSE06 agree reasonably well with experimental values. The calculated dielectric constants, although showing significant discrepancies with experimental values, confirm that these oxynitrides are promising for applications as dielectric materials. Our work provides a theoretical perspective to the anion order in perovskite oxynitrides and could possibly offer valuable information for further tuning anion order and therefore their properties as functional materials.

## Conflicts of interest

There are no conflicts to declare.

## Supplementary Material

RA-010-D0RA03681A-s001
