# Supplementary material for: Anion order in perovskite oxynitrides AMO2N (A = Ba, Sr, Ca; M = Ta, Nb): a first-principles based investigation
Source: RSC Adv. 2020 Jun 25;10(41):24410–8. doi: 10.1039/d0ra03681a (PMC9055086; doi:10.1039/d0ra03681a)
Supplement: RA-010-D0RA03681A-s001 [file RA-010-D0RA03681A-s001.pdf]

Electronic Supplementary Material (ESI) for RSC Advances.
This journal is © The Royal Society of Chemistry 2020

Supplementary information

Anion order in perovskite oxynitrides AMO_2N (A = Ba, Sr, Ca; M = Ta, Nb): a first-principles based investigation

Xi Xu and Hong Jiang*
Beijing National Laboratory for Molecular Sciences, College of Chemistry and Molecular Engineering, Peking University, 100871 Beijing, China.
E-mail: jianghchem@pku.edu.cn

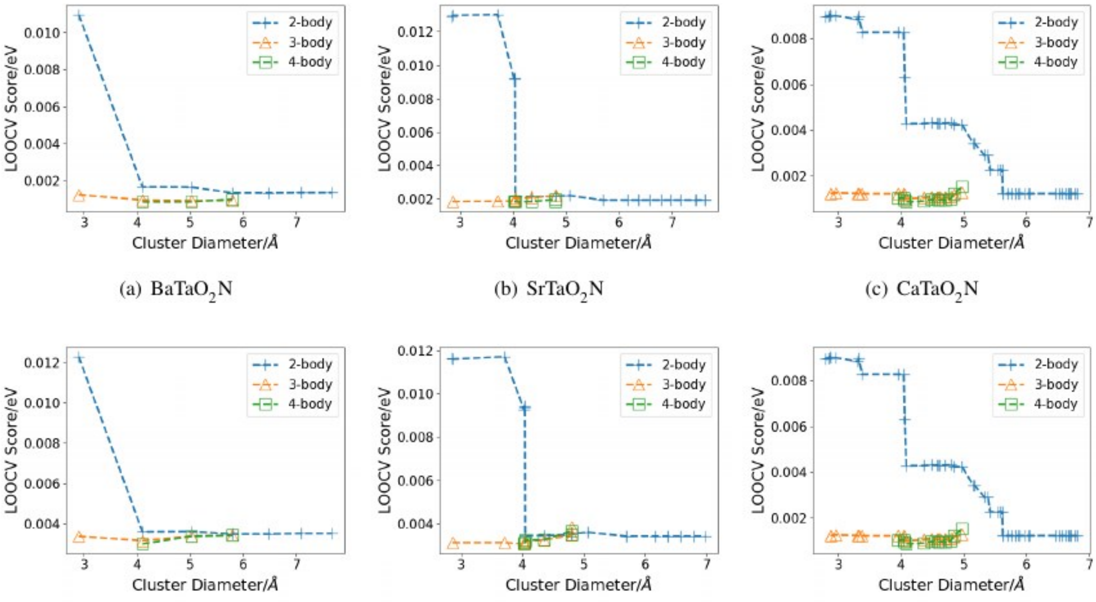

(a) $BaTaO_2N$ (b) $SrTaO_2N$ (c) $CaTaO_2N$

(d) $BaNbO_2N$ (e) $SrNbO_2N$ (f) $CaNbO_2N$

Figure S1: The leave-one-out cross-validation (LOOCV) scores with respect to different cluster expansion models of perovskite oxynitrides.

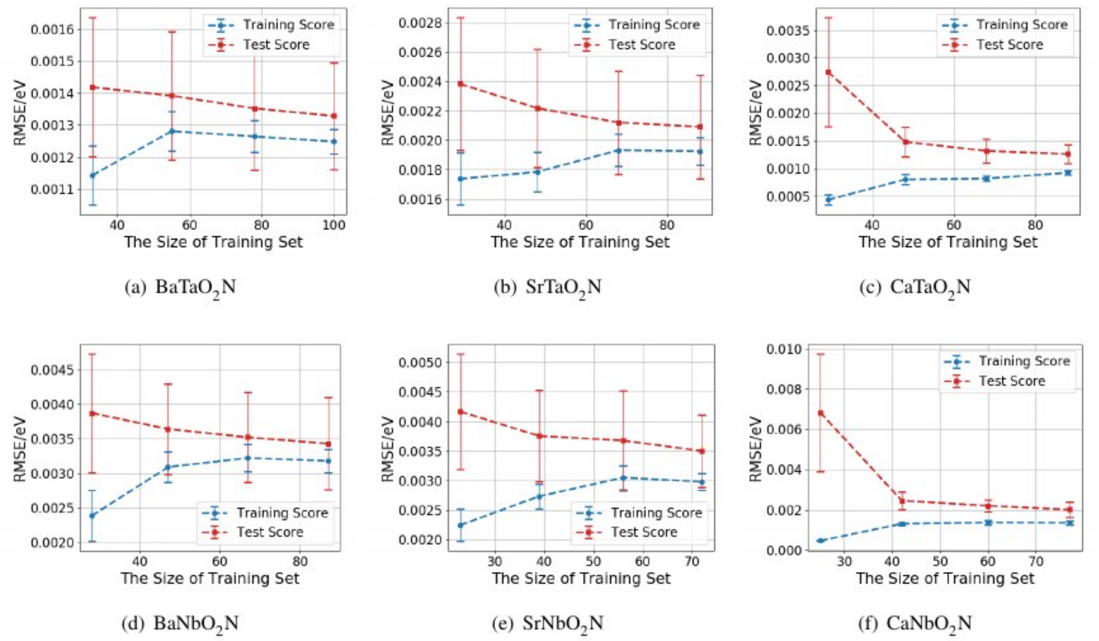

(a) $BaTaO_2N$ (b) $SrTaO_2N$ (c) $CaTaO_2N$

(d) $BaNbO_2N$ (e) $SrNbO_2N$ (f) $CaNbO_2N$

Figure S2: Learning curves for the optimal cluster expansion models with least-squares fitting of ECIs.

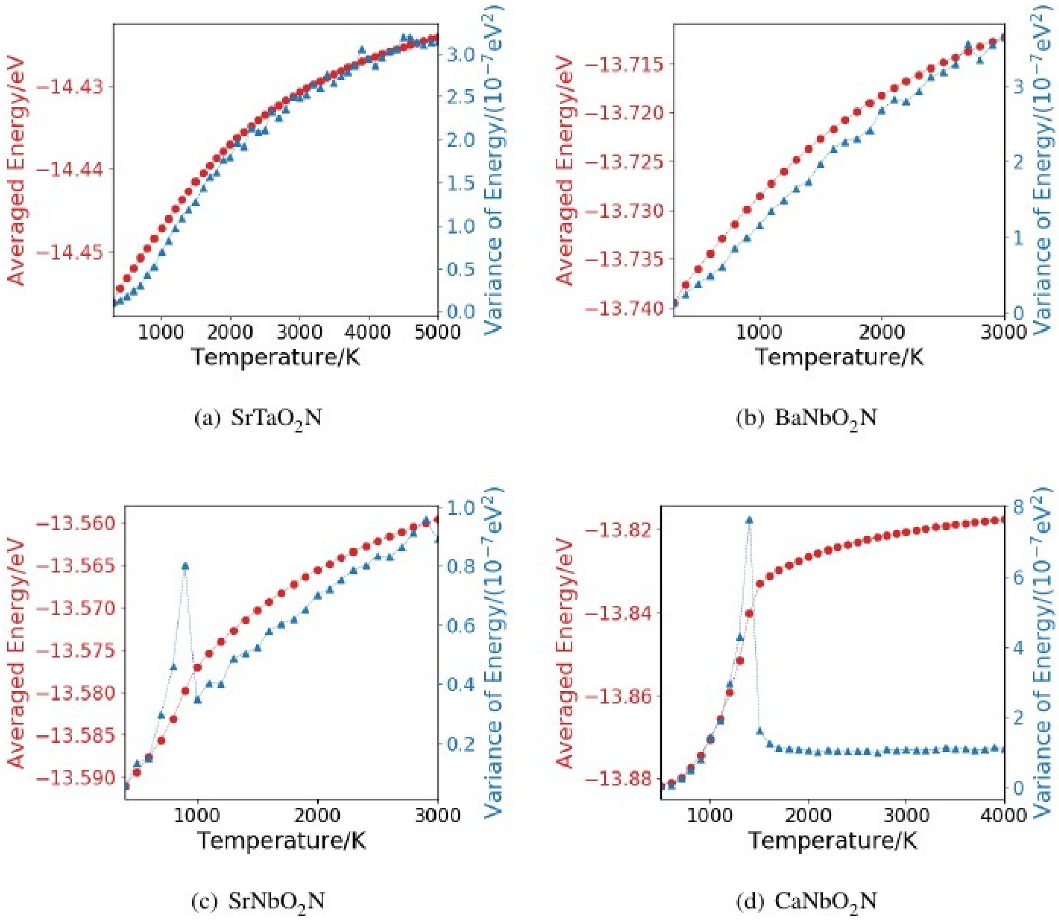

(a) $SrTaO_2N$ (b) $BaNbO_2N$

(c) $SrNbO_2N$ (d) $CaNbO_2N$

Figure S3: Statistically averaged energies and variance of Monte Carlo simulations for

$SrTaO_2N$, $BaNbO_2N$, $SrNbO_2N$ and $CaNbO_2N$ respectively.

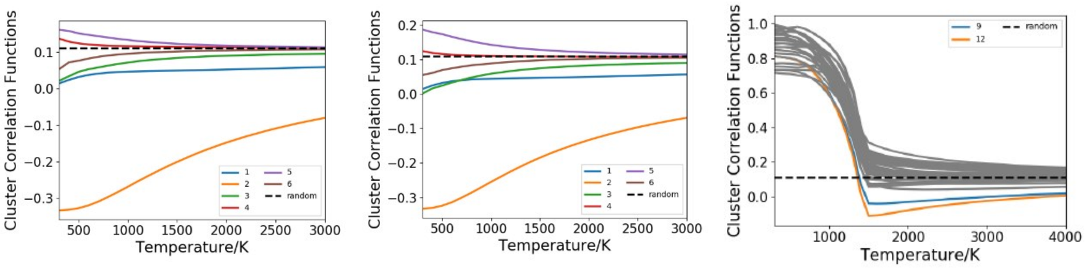

(a). $BaTaO_2N$ (b) $BaNbO_2N$ (c) $CaNbO_2N$

Figure S4: Cluster correlation functions (CCFs) in $BaTaO_2N$, $BaNbO_2N$ and $CaNbO_2N$ calculated by Monte Carlo simulations.

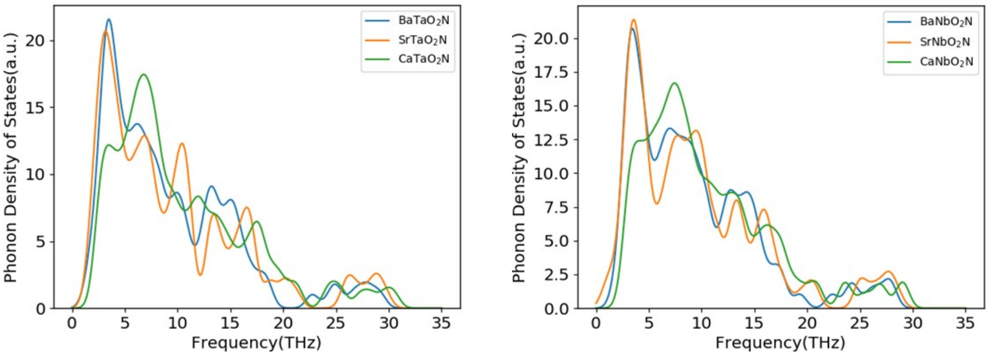

Figure S5: Phonon density of states for the SQoSs of AMO_2N (A = Ba, Sr, Ca; M = Ta, Nb).
